# The choice of reference point for computing sagittal plane angular momentum affects inferences about dynamic balance

**DOI:** 10.7717/peerj.13371

**Published:** 2022-05-12

**Authors:** Chang Liu, Sungwoo Park, James Finley

**Affiliations:** 1 Department of Biomedical Engineering, University of Southern California, Los Angeles, CA, United States of America; 2 School of Engineering and Applied Sciences, Harvard University, Cambridge, MA, United States of America; 3 Division of Biokinesiology and Physical Therapy, University of Southern California, Los Angeles, CA, United States of America

**Keywords:** Angular momentum, Balance, Gait, Locomotion, Reactive control, Stability

## Abstract

**Background:**

Measures of whole-body angular momentum in the sagittal plane are commonly used to characterize dynamic balance during human walking. To compute angular momentum, one must specify a reference point about which momentum is calculated. Although biomechanists primarily compute angular momentum about the center of mass (CoM), momentum-based controllers for humanoid robots often use the center of pressure. Here, we asked if the choice of the reference point influences interpretations of how dynamic balance is controlled in the sagittal plane during perturbed walking.

**Methods:**

Eleven healthy young individuals walked on a dual-belt treadmill at their self-selected speed. Balance disturbances were generated by treadmill accelerations of varying magnitudes and directions. We computed angular momentum about two reference points: (1) the CoM or (2) the leading edge of the base of support and then projected it along the mediolateral axes that pass through either of the reference points as the sagittal plane angular momentum. We also performed principal component analysis to determine if the choice of reference point influences our interpretations of how intersegmental coordination patterns contribute to perturbation recovery.

**Results:**

We found that the peak angular momentum was correlated with perturbation amplitude and the slope of this relationship did not differ between reference points. One advantage of using a reference point at the CoM is that one can easily determine how the momenta from contralateral limbs, such as the left and right legs, offset one another to regulate the whole-body angular momentum. Alternatively, analysis of coordination patterns referenced to the leading edge of the base of support may provide more insight into the inverted-pendulum dynamics of walking during responses to sudden losses of balance.

## Introduction

Human locomotion is inherently unstable due to the small base of support, long single-limb support times, and sensorimotor transmission delays (Winter, 1995; Woollacott & Tang, 1997). During walking, the body’s center of mass (CoM) routinely moves beyond the base of support (BoS) during the single support phase, and this poses a challenge for maintaining dynamic balance. One way to generate corrective responses to maintain balance in response to both internal and external perturbations is through the reactive control of balance, which involves the use of feedback about the body’s state to generate balance correcting responses (Patla, 1993; Tang, Woollacott & Chong, 1998). For example, people can actively rotate both the upper and lower limbs in a coordinated way to counteract the body’s rotation toward the ground (Hof, 2007).

One common measure to capture dynamic balance is whole-body angular momentum. This measure reflects the net contribution of all body segments to the body’s rotation about a specified reference point. To compute angular momentum in the sagittal plane, biomechanists typically choose to calculate angular momentum about the CoM and then project it on the mediolateral axis passing through the CoM (Bennett et al., 2010; Herr & Popovic, 2008; Thielemans, Meyns & Bruijn, 2014). Angular momentum projected along this axis is highly regulated during normal human locomotion as the peak-to-peak range of angular momentum is much smaller than the angular momentum of single segments due to momentum cancellation between the limbs (Herr & Popovic, 2008; Popovic, Hofmann & Herr, 2004). The range of angular momentum is also kept small when walking at different speeds (Bennett et al., 2010), walking with different step lengths (Thielemans, Meyns & Bruijn, 2014), or during stair ascent/descent (Silverman et al., 2014). Additionally, whole-body angular momentum computed about the CoM has been used to assess balance impairments among populations with an increased risk of falls (Honda et al., 2019; Silverman & Neptune, 2011). For example, people post-stroke have a larger peak-to-peak range of angular momentum during the non-paretic step compared to controls (Honda et al., 2019), and larger magnitudes of angular momentum during walking are associated with worse performances in clinical balance assessments (Nott, Neptune & Kautz, 2014; Park et al., 2021). Angular momentum can also capture changes in body dynamics in the sagittal plane during trips or slips which are the most prevalent cause of falls in older adults (Berg et al., 1997). For example, trips or slips typically result in a sharp increase in momentum from that measured during unperturbed walking (Liu, De Macedo & Finley, 2018; Liu & Finley, 2020; Martelli et al., 2013; Pijnappels, Bobbert & Van Dieën, 2004; Pijnappels, Bobbert & van Dieën, 2005; Potocanac et al., 2014). These deviations in angular momentum capture the features of body rotation that could lead to fall during perturbations.

While biomechanists typically compute angular momentum about the CoM (Herr & Popovic, 2008; Liu, De Macedo & Finley, 2018; Martelli et al., 2013), computing angular momentum about the leading edge of the BoS may provide additional information about whole-body dynamics (Chiovetto, Sternad & Giese, 2018). Referencing angular momentum to a point that is approximately located at the border of the foot’s contact surface with the ground may be best for capturing the inverted pendulum dynamics of walking (Cavagna, Heglund & Taylor, 1977; Kuo, Donelan & Ruina, 2005; Lee & Farley, 1998). This is because the superior segments with larger mass, such as the trunk and the head, would likely have a dominating contribution to whole-body angular momentum given their distance from the reference point (Gaffney et al., 2017). Additionally, roboticists compute angular momentum about the foot contact point to account for the inverted pendulum dynamics of walking and predict the feasible range of subsequent foot placement positions that will allow the biped to continue steady-state walking (Millard et al., 2009; Wight, Kubica & Wang, 2008). Thus, there are tradeoffs in the insights about how humans control balance during walking that depend on the choice of reference point used to compute angular momentum.

Dimensionality reduction techniques, such as principal component analysis (PCA), are commonly used to capture how multiple limb segments are coordinated during reactive control of angular momentum (Aprigliano et al., 2017; Chiovetto, Sternad & Giese, 2018; Liu & Finley, 2020; Martelli et al., 2013). Performing PCA on segmental angular momenta can be used to infer the degree of segmental cancellation of angular momenta during walking. For example, as the right and left limbs rotate in anti-phase in the sagittal plane, PCA can capture momentum cancellation between the left and right limbs in the sagittal plane about CoM by showing that the weighting coefficients of the left and right limbs have opposite signs in the extracted coordination patterns (Herr & Popovic, 2008; Liu & Finley, 2020). During responses to perturbations, the degree of segmental cancellation decreases so that angular momentum increases sharply. However, it is unclear if extracting segmental coordination patterns using measures of angular momentum referenced to the edge of the BoS would provide similar interpretations about how humans coordinate body segments in response to perturbations.

The objective of this exploratory study is to determine whether using different reference points to compute angular momentum would influence interpretations of dynamic balance control strategies during treadmill-elicited perturbations. To this end, we imposed both posteriorly- and anteriorly-directed perturbations on a dual-belt treadmill. We then used PCA to determine if the segmental coordination patterns observed differ when angular momentum is computed with respect to the CoM *versus* the edge of BoS. We then determined whether our interpretations about how people coordinate their segments to restore angular momentum during perturbation responses differ if we compute angular momentum with respect to different reference points. Ultimately, our findings will extend our understanding of how the healthy central nervous system coordinates intersegmental dynamics to maintain balance during perturbation responses during walking.

## Materials & Methods

### Participant characteristics

A total of 11 healthy young individuals (5M, 27 ± 3yrs old, 67.9 ± 17.2 kg, self-selected walking speed = 1.0 ± 0.1 m/s) with no musculoskeletal or gait impairments participated in this study. Participants self-reported the right side as their dominant limb when asked which leg they would use to kick a ball. The study was approved by the Institutional Review Board (#HS-18-00533) at the University of Southern California, and all participants provided written informed consent before participating. All aspects of the study conformed to the principles described in the Declaration of Helsinki.

### Experimental protocol

Participants walked on an instrumented, dual-belt treadmill with force plates underneath (Bertec, USA) for six separate trials and reacted to accelerations of the treadmill belts throughout the experiment. Their self-selected walking speed was first determined using a two-alternative forced-choice staircase method (Dal et al., 2010; Farrens, Lilley & Sergi, 2020; Garcia et al., 1998). We first determined the participants’ overground walking speed using the 10-meter walk test (Bohannon, 1997). Then we used 50% of their overground walking speed as a starting point to determine their self-selected walking speed on a treadmill. Treadmill speed was first incrementally increased by 0.02 m/s, and the participant was asked if they were walking at their self-selected speed after each change until the participant verbally confirmed this was their walking speed. Then we incrementally decreased the treadmill speed by 0.02 m/s from a speed that was 0.04 m/s faster than the speed they just chose until the participant verbally confirmed again. We repeated the process twice using a custom Matlab program (Matlab 2020b, Mathworks, USA). We computed the final self-selected walking speed by averaging the four walking speeds the participant identified. For the first trial, participants walked on the treadmill for five minutes at their self-selected walking speed. We informed the participants that no treadmill-induced perturbations would occur during this trial. Then, for five subsequent trials (two participants completed only four perturbation trials), we informed the participants that treadmill perturbations of different magnitude and direction would occur at random foot strikes. Each trial consisted of a total of 24 perturbations with 12 on each belt. The perturbations had magnitudes at −0.5 m/s, −0.4 m/s, −0.3 m/s, 0.3 m/s, 0.5 m/s, and 0.7 m/s, where positive values indicated increases in speed relative to the participant’s self-selected walking speed, and negative values corresponded to reductions in the participant’s self-selected walking speed. The order of these perturbations was randomized. Foot strike was computed as the point when vertical ground reaction forces reached 80 N. Each perturbation was remotely triggered by customized Matlab code. The perturbation was characterized by a trapezoidal speed profile in which the treadmill accelerated at liftoff to the target belt speed at an acceleration of 3 m/s^2^ (or −3 m/s^2^ if the target speed was less than their walking speed), held this speed for 0.7 s, and then returned to the participant’s self-selected walking speed at an acceleration of −3 m/s^2^ (or 3 m/s^2^). The perturbations were randomly triggered to occur within a range of 15 to 25 steps after the previous perturbation to prevent participants from anticipating perturbation timing. We also selected this range of steps to provide participants with sufficient time to reestablish their baseline walking pattern.

### Data acquisition

A ten-camera motion capture system (Qualisys AB, Gothenburg, Sweden) recorded 3D marker kinematics at 100 Hz and ground reaction forces at 1000 Hz. We placed a set of 14 mm spherical markers on anatomical landmarks to create a 13-segment, full-body model (Havens, Mukherjee & Finley, 2018; Song et al., 2012). We placed marker clusters on the upper arms, forearms, thighs, shanks, and the back of heels. Marker positions were calibrated during a five-second standing trial. We removed all joint markers after the calibration.

### Data processing

We post-processed the kinematic and kinetic data in Visual3D (C-Motion, Rockville, MD, USA) and Matlab 2020b (Mathworks, USA) to compute variables of interest. Marker positions and ground reaction forces were lowpass filtered by 4th order Butterworth filters with cutoff frequencies of 6 Hz and 20 Hz, respectively (Kurz, Arpin & Corr, 2012; Reisman et al., 2009; Winter, 2009). We defined foot strike as the point when the vertical ground reaction force reached 80N. We also examined the timing of perturbations relative to foot strike post-hoc to remove the perturbations that began more than ∼150 ms after the foot-strike (Buurke et al., 2020). We included a median of 10 (interquartile range: 2) perturbations for each perturbation amplitude per side for each participant. We categorized pre-perturbation (Pre-PTB) steps as the two steps before the perturbation occurred and perturbation (PTB) steps as the step during which the perturbation was applied. For treadmill perturbations that elicited a backward loss of balance, some participants generated an aborted step during the PTB steps. We defined an aborted step as one in which the trailing leg lifted off the treadmill and landed again after the perturbation was initiated without the contralateral perturbed leg being lifted off of the treadmill (Bhatt, Wening & Pai, 2005). In this case, we defined the perturbation steps to end with trailing foot landing on the treadmill. We also focused our analysis on angular momentum in the sagittal plane as this was the plane in which the most prominent changes in body rotational behavior were observed.

### Segmental angular momentum and whole-body angular momentum

We created a 13-segment, whole-body model in Visual3D and calculated the angular momentum of each segment about two different reference points (Liu & Finley, 2020). Segmental angular momenta (${L}_{s}^{\mathrm{i}}$) captured changes in the rotational and translational behavior of each body segment during treadmill-elicited perturbations (Liu & Finley, 2020). The model was similar to previously described in Liu & Finley (2020). Specifically, the model included the following segments: head, thorax, pelvis, upper arms, forearms, thighs, shanks, and feet. We modeled the limb segments’ mass based on anthropometric tables (Dempster, 1955), and the segment geometry based on the description in Hanavan (1964). Segmental angular momentum was computed using Eq. (1) (Silverman & Neptune, 2011).

(1) \begin{eqnarray*}{L}_{s}^{\mathrm{i}}={m}_{i} \left( {r}_{Ref-i}^{i}\times {v}_{Ref-i}^{i}\text{} \right) \mathrm{~ }+{I}^{i}{\omega }^{i}.\end{eqnarray*}

Here, ${L}_{s}^{\mathrm{i}}$ is the segmental angular momentum, *m*_*i*_ is segmental mass, *r*_*Ref*−*i*_ is the displacement from the segment’s CoM to the reference axis, *v*_*Ref*−*i*_ is the velocity of each segment’s CoM relative to the reference axis, *I*^*i*^ is the segmental moment of inertia about the principal axes of the segment, *ω*^*i*^ is segmental angular velocity, and the index *i* corresponds to individual limb segments. Sagittal plane angular momentum was defined as the projection of angular momentum on the mediolateral axis passing through either of the two reference points: (1) the CoM (L_CoM_) or (2) the leading edge of the base of support (BoS) of the stance limb as estimated by a marker on the first phalanx (L_BoS_). Both axes were defined as positive to the person’s right.

Whole-body angular momentum was calculated as the sum of all segmental angular momenta using Eq. (2).

(2) \begin{eqnarray*}L= \frac{\sum _{\mathbi{i}}{L}_{s}^{\mathrm{i}}}{MVH} .\end{eqnarray*}

We normalized momentum by the participant’s mass (*M*), self-selected treadmill velocity (*V*), and the participant’s height (*H*) (Eq. (2)) following previous literature (Herr & Popovic, 2008; Honda et al., 2019; Liu & Finley, 2020). We used the maximum and minimum value of L_COM_ and L_BoS_ during the perturbation step to quantify the effect of perturbations (Martelli et al., 2013). The maximum value of L indicated peak backward rotation and the minimum value of L indicated peak forward rotation.

### Principal component analysis (PCA)

We used PCA to extract intersegmental coordination patterns for each step cycle (Chiovetto, Sternad & Giese, 2018; Liu & Finley, 2020; Martelli et al., 2013). Before performing PCA, we first time normalized the segmental angular momenta to 100 points for each step cycle. Then, for each participant, we created seven *L*_*s*_ matrices, including one matrix for Pre-PTB steps and six matrices corresponding to the set of perturbation amplitudes used in the study. We only included perturbations of the right (dominant) side for this analysis. The *L*_*s*_ matrices had dimensions of n_steps*100 rows and 13 columns, one for each segment. We then standardized each matrix to have zero mean and performed PCA to extract subject-specific coordination patterns using the *prcomp* function in R (version 3.6.1). Using PCA, we decomposed the segmental angular momenta data into (1) a weighting coefficient matrix consisting of PCs ordered according to their variance accounted for (VAF) and (2) time series scores which represented the activation of each PC throughout the step cycle. PCs were ranked by how much variance in the data was explained by each of them. We retained the number of PCs necessary to account for at least 90% (95% CI) of variance in *L*_*s*_.

### Comparison of intersegmental coordination patterns

We investigated if inferred segmental coordination patterns differed when PCs were extracted from segmental angular momenta referenced to the body’s CoM *versus* the leading edge of the BoS. We first sorted the PCs so that pairs of similar PCs were aligned across participants (Chiovetto, Sternad & Giese, 2018; Torres-Oviedo & Ting, 2010). Each participant’s PCs were matched to that of one reference participant by calculating the scalar product (r) of the two PCs as this is a common method to compare the similarity between vectors in a high-dimensional space. The scalar product of the unit vectors was between 0 (orthogonal and most dissimilar) and 1 (parallel and identical). The PC that was the most similar to the first PC of the reference participant (scalar product closer to 1) was assigned as the first PC for the participant. The PCs of the first best-matching pair were then removed from the PCs and we repeated the same procedure to match the next pair of PCs and so forth.

Then, we assessed the similarity between PCs referenced to two different points by computing the scalar product to match the most similar PCs extracted from the sagittal plane segment angular momenta referenced to the CoM and the edge of BoS. This analysis allowed us to examine whether the coordination patterns referenced to two points were similar. We first identified the most similar PC_BoS_ to PC1_CoM_ for each subject for each perturbation level. Then we repeated the same process for PC1_BoS_. A pair of PCs were considered “similar” if *r* > 0.684, which corresponded to a significance level of *p* < 0.01 for vectors in a 13-dimensional space (since we have 13 segments). Otherwise, a pair of PCs was considered ‘dissimilar’.

### Statistical analysis

We first determined if the minimum and maximum angular momentum during the perturbation steps were associated with the perturbation amplitude (Amplitude) and the side of the perturbation (Side). We performed linear mixed-effects regression analyses in Matlab to examine the relationship between the independent variables Amplitude, Side, and interaction between Amplitude and Side, and each dependent variable of peak angular momentum (Max L_CoM_, Max L_CoM_, Min L_BoS_, Min L_BoS_). For each regression model, we determined if a model with random effects provided a better fit than a model with only fixed effects by comparing both models using Akaike Information Criterion (AIC) (Akaike, 1981). We selected the model with a lower AIC. Based on this analysis, we included random effects in all models to account for the individual differences between subjects.

We also examined whether there were differences in the variance explained by the PCs by performing a two-sample Welch’s *t*-test to compare the variance explained for each reference point. To examine the similarity between PCs extracted from segmental angular momenta referenced to the two points, we tested if the scalar product between the most similar pairs of PC_BoS_ and the first PC_CoM_ and pairs of PC_CoM_ and the first PC_BoS_ was greater than the critical r value using a one-tailed *t*-test. A scalar product that was equivalent to or higher than the critical value indicated that the two PCs were statistically similar (Allen et al., 2017). These analyses were performed in RStudio (3.6.2). We checked normality using the Shapiro–Wilk normality test. If any of the data were not normally distributed, we log-transformed the data to ensure that they were normally distributed before performing the two-sampled Welch’s *t*-test. We reported normally distributed values as mean ± standard deviation of the corresponding mean and non-normally distributed data as median with interquartile range (IQR), [25% IQR, 75% IQR]. Significance was set at *p* < 0.05 level.

## Results

### Changes of whole-body angular momentum in response to treadmill perturbations

**Figure 1:**
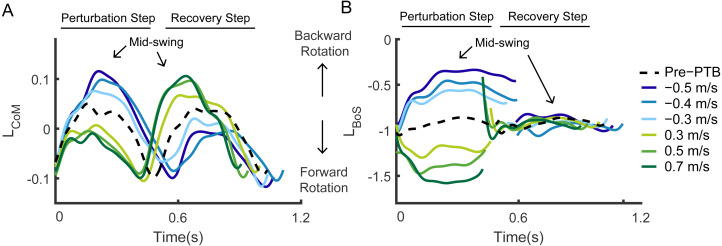
Whole-body angular momentum in the sagittal plane with respect to two reference points during pre-perturbation, perturbation, and the following recovery steps for one representative participant. Whole-body angular momentum referenced to the (A) CoM and (B) edge of BoS. Dashed lines: angular momentum referenced to the edge of BoS and CoM during pre-perturbation walking. Colored lines: angular momentum during perturbation steps and the first recovery steps. Arrows indicate the approximate mid-swing point for each step.

During steps before the perturbations, L_CoM_ in the sagittal plane was most negative during the transition from the swing phase to the stance phase when the peak forward momentum occurred (Fig. 1A). Then, L_CoM_ increased to become positive until mid-swing before becoming negative again during late stance. The peak backward momentum occurred during mid-swing of each step, which corresponds to ∼25% and ∼75% of the gait cycle. L_BoS_ was also most negative during the transition from the swing phase to the stance phase (Fig. 1B). Participants increased L_BoS_ until mid-swing and then momentum became more negative during late stance.

Measures of angular momentum varied in response to changes in perturbation magnitude. During treadmill accelerations, angular momentum relative to both reference points became more negative as the body rotated forward (Fig. 1). Participants then generated more positive angular momentum during the recovery step and initiated backward rotation to recover from the perturbations. During treadmill deceleration, angular momentum initially became more positive as the body rotated backward (Fig. 1). Similar to what was observed during the pre-perturbation steps, the peak backward angular momentum occurred around mid-swing and the peak forward angular momentum occurred during the transition from swing to stance phase during the treadmill perturbations.

### Effects of perturbation amplitude on angular momentum

**Figure 2:**
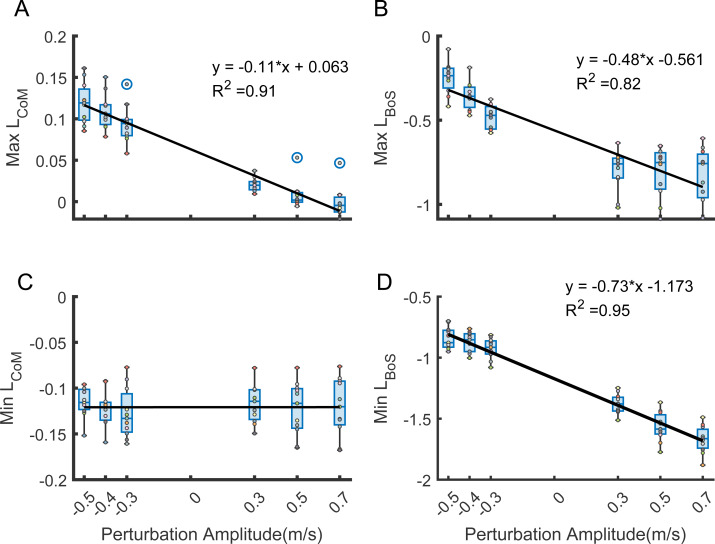
Association between perturbation amplitude and peak whole-body angular momentum referenced to CoM and the edge of BoS across participants (*N* = 11). The black lines represent the fixed effects from the mixed-effect model. *R*^2^ values measure the goodness of fit of each mixed-effect model. (A–B) Maximum L_CoM_ and L_BoS_ were negatively correlated with perturbation amplitude. (C) Minimum L_CoM_ was not correlated with perturbation amplitude. (D) Minimum L_BoS_ were negatively correlated with perturbation amplitude. Each dot represents one participant.

Treadmill decelerations were associated with increases in backward angular momentum while treadmill accelerations were associated with forward increases in angular momentum. We did not find any significant effects of Side or an interaction between Side and Amplitude on any of the dependent variables and thus we combined perturbations on both sides for this analysis. Peak backward angular momentum in the sagittal plane was negatively associated with perturbation amplitude for L_CoM_ (F (1,128) = 432, *p* < 0.001) and L_BoS_ (F (1, 128) = 228, *p* < 0.001) during the perturbation steps (Figs. 2A & 2B). The peak forward angular momentum during the perturbation steps was negatively correlated with the perturbation amplitude for L_BoS_ (F(1,128) = 1127, *p* < 0.001) but not for L_CoM_ (*p* = 0.99) (Figs. 2C & 2D).

### Variance accounted for (VAF) by PCs

**Figure 3:**
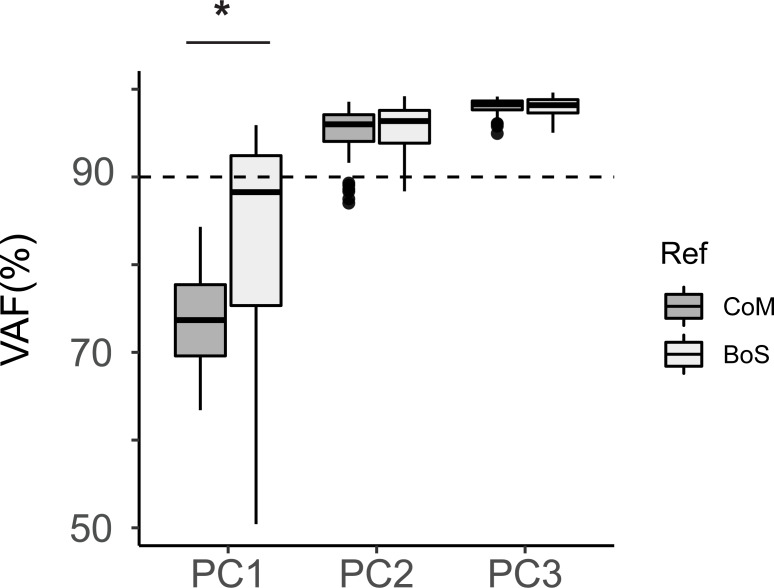
Cumulative VAF for the first three PCs across all levels of perturbations and pre-perturbation steps. Dashed horizontal line indicated the 90% variance cut-off. Dark shaded: angular momentum referenced to the CoM; lightly shaded: angular momentum referenced to the edge of BoS. (*p < 0.001).

On average across all perturbation levels, two principal components accounted for more than 90% of the variance in segmental angular momentum (Fig. 3). PC1 explained 74 ± 6% of the variance, PC2 explained 22 ± 5% of the variance, and PC3 accounted for 3 ± 2% of the variance for angular momentum referenced to the CoM. For segmental angular momentum referenced to the BoS, PC1 explained 83 ± 12% of the variance, PC2 explained 12 ± 10% of the variance, and PC3 accounted for 2 ± 1% of the variance. Therefore, we retained two PCs for segmental angular momentum referenced to both points. The first principal component explained more variance for angular momentum referenced to the edge of BoS than to the CoM (t(108) = −6.5, *p* < 0.001). However, the cumulative variance explained for angular momentum was similar for the first two PCs (t(151) = −1.01, *p* = 0.31) and first three PCs (t(143) = 0.5, *p* = 0.62).

### Segmental coordination patterns differed based on the reference point chosen for angular momentum

**Figure 4:**
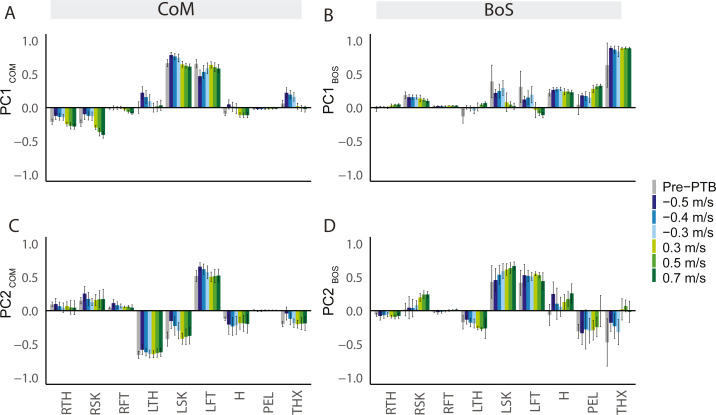
Principal components (PC) extracted from segmental angular momenta referenced to two different points during pre-perturbation and perturbation steps (*N* = 11). PCs referenced to the CoM (A, C). PCs extracted from segmental angular momenta referenced to the edge of BoS (B, D). The segments include: RTH (right thigh), RSH (right shank), RFT (right foot), LTH (left thigh), LSH (left shank), LFT (left foot), H (head), PEL (pelvis), THX (thorax). Average PC weights for arm segments (forearms and upper arms) are below 0.1, so they are not included in this figure to improve clarity. Error bars represented standard deviation.

We only included perturbations of the right (dominant) leg for the principal component analysis and thus, we refer to the right limb as the ipsilateral perturbed limb and the left limb as the contralateral limb. For L_CoM_, contributions from the lower extremities were typically dominant in the first PC, while contributions from the arms, pelvis, trunk, and head were negligible (Fig. 4A). During perturbed steps, the contralateral limb was in the swing phase and generated more positive momentum about the body’s CoM while the ipsilateral perturbed limb generated negative momentum. Thus, the signs of the weighting coefficients for the contralateral leg segments (left thigh, shank, and foot) were opposite to the weighting coefficients of the ipsilateral (right) leg segments. Overall, the first PC captured the opposing momenta of the two legs resulting from differences in the direction of rotation relative to the body’s CoM. For PC2_CoM_, the weighting coefficients for distal lower extremity segments were also larger than the weighting coefficients for proximal segments, although the coefficients for the thorax and head increased compared to that in PC1_CoM_ (Fig. 4C).

For L_BoS,_ contributions from the proximal segments such as pelvis, thorax, and head, were typically dominant in the PC1_BoS_ (Fig. 4B), while contributions from the lower extremities were much lower than what we observed in PC1_CoM_. For PC1_BoS,_ the weighting coefficients of the upper segments have same signs, indicating that the angular momentum for the upper body segments were positively correlated. For PC2_BoS,_ contributions from the contralateral leg segments were dominant while the contributions from the proximal segments were smaller compared to that in PC1_BoS_ (Fig. 4D). Overall, PC1_BoS_ captured the variance in segmental angular momenta due to motion of proximal segments while PC2_BoS_ captured the variance of segmental angular momenta due to motion of the contralateral swing leg.

**Figure 5:**
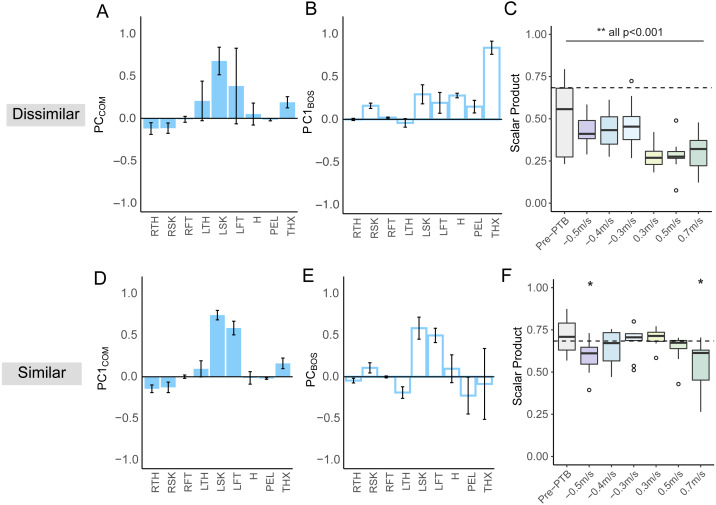
Dissimilar and similar PCs and extracted from segmental angular momenta referenced to CoM and the leading edge of BoS and the scalar products comparing the similarity between the two PCs. (A) PC1_BoS_ and its matched PC_CoM_(B) during perturbation steps with −0.3 m/s speed change. Error bars represented standard deviation. (C) Boxplot shows the scalar product between the PC1_BoS_ and the matched PC_CoM_ extracted from segmental angular momentum during pre-perturbation steps and perturbation steps at different levels of speed change (**p < 0.001). (D) PC1_CoM_ and its matched PC_BoS_ (E) during perturbation steps with −0.3 m/s speed change. Error bars represented standard deviation. (F) Boxplot shows the scalar product between the PC1_CoM_ and the matched PC_BoS_ extracted from segmental angular momentum during pre-perturbation steps and perturbation steps at different levels of speed change. (*p < 0.05). Horizontal dashed line indicates the critical r value = 0.684 for the two PCs to be similar in the 13-dimensional space. Asterisks indicate that the scalar products were lower than the critical r value.

Lastly, we compared the intersegmental coordination patterns (*i.e.,* PCs) extracted from angular momentum with respect to two different reference points. PC1_BoS_ was not found to be similar to any of the PCs computed related to the center of mass. The scalar products between PC1_BoS_ and the PC_CoM_ across all steps were all significantly lower than *r* = 0.684 (*p* < 0.001) (Fig. 5C). When a coordination pattern that was extracted relative to the base of support was matched with PC1_CoM_, it was typically PC2_BoS_. These matched pairs of PCs were generally found to be similar as the scalar products between the PC1_CoM_ and the matched PC_BoS_ were equivalent to or higher than the critical r value. Exceptions only occurred when the speed change was −0.5 m/s (t(10) = −3.3, *p* = 0.004) and 0.7 m/s (t(10) = −3.4, *p* = 0.003) (Fig. 5F). In summary, PC_CoM_ and PC_BoS_ typically had one shared coordination pattern and one dissimilar coordination pattern.

## Discussion

The objective of this study was to investigate whether computing angular momentum in the sagittal plane with respect to different reference points would influence our interpretations of dynamic balance control during treadmill-induced perturbations. We found that peak backward angular momentum during the perturbed steps was positively associated with perturbation amplitude regardless of the reference point used to define angular momentum. In addition, the low-dimensional intersegmental coordination patterns extracted when referenced to the BoS and CoM had one similar component and one dissimilar component during perturbation steps, indicating that these methods provide complementary information about how healthy people coordinate their segments to maintain angular momentum during perturbation responses.

The observation that the directions of the association between perturbation amplitude and the peak backward L_CoM_ or L_BoS_ were the same between methods reflects the parallel axis theorem. We observed that participants’ peak backward angular momentum in the sagittal plane was negatively associated with changes in perturbation speed suggesting that participants fell forward more with larger increases in treadmill speed and fell backward more with larger reductions in treadmill speed. The intercept and slope magnitudes of the two regression analyses relating peak backward angular momentum and perturbation amplitude were different due to the large distance between CoM and the leading edge of BoS. On the other hand, the peak forward angular momentum during the perturbation steps was only negatively correlated with the perturbation amplitude for L_BoS_ but not for L_CoM_. The lack of an association between peak forward L_CoM_ and perturbation amplitude was likely because the minimum values usually occurred at the foot strike before the perturbations had any effect on body dynamics.

The whole-body response to perturbations during normal walking and perturbation steps can be characterized by low-dimensional patterns that capture the coordination between segments. We identified how the body segments covaried to control angular momentum to avoid falling by extracting intersegmental coordination patterns from segmental angular momentum. There was considerable variability in the VAF for the PC1_BoS_ (Fig. 3). This high variability is likely because the VAF for the PC1_BoS_ were not consistent between forward and backward perturbations. During forward perturbation steps, variance in L_BoS_ is dominated by the first PC that describes the inverted pendulum dynamics of the upper body segments. However, during the backward perturbation steps, variance in L_BoS_ may be equivalently explained by the coordination patterns that describe both inverted pendulum mechanics as well as the inter- and intra-segment covariation in the lower extremities so that the first PC’s VAF is lower than that during forward perturbation steps.

Nevertheless, both the first two PC_CoM_ and PC_BoS_ explained more than 90% of the variance of the segmental angular momenta referenced to the CoM and the edge of BoS. This is consistent with a previous finding that angular momentum referenced to both CoM and the contact point in the mediolateral plane during beam walking could be characterized by low dimensional segmental coordination patterns (Chiovetto, Sternad & Giese, 2018). Taken together, these results suggest that changing the reference point of angular momentum calculation does not alter the hypothesis that the coordination of body segments under balance challenging conditions can be represented in a lower dimensionality.

In addition, we observed several differences between the coordination patterns extracted from sagittal plane angular momentum with respect to different reference points. First, the trunk, pelvis, and head contributions to whole-body angular momentum were small when referenced to CoM as the distances between these segments and the CoM were minimal. In contrast, when referenced to the BoS, these segments that were far away from the BoS dominated the PC weights, which was in line with the previous studies (Chiovetto, Sternad & Giese, 2018; Gaffney et al., 2017). Controlling the rotation of upper-body segments to maintain angular momentum in the sagittal plane is important as the upper-body segments have high inertia, especially the trunk. Both abdominal and back muscles need to respond rapidly following trips to reduce the excessive forward rotation in the sagittal plane (Van Der Burg, Pijnappels & Van Dieën, 2005). Therefore, analyzing coordination patterns referenced to the leading edge of the base of support may provide more insights into how the upper body is controlled in response to losses of balance.

Secondly, the coordination pattern extracted in PC1_CoM_ was useful for inferring how the contributions from the lower limbs counteracted disturbances to whole-body angular momentum during unexpected perturbations. PC1_CoM_ indicated that the swing leg and perturbed leg generated angular momentum in opposite directions about the CoM. Moving contralateral limb segments in anti-phase helps to minimize changes in whole-body angular momentum. These results were consistent with previous literature demonstrating that the perturbed leg and the contralateral swing leg were in anti-phase during walking and in response to perturbations (Herr & Popovic, 2008; Liu & Finley, 2020; Martelli et al., 2013). In contrast, the PC_BoS_ that were most similar to PC1_CoM_ did not provide the same information about leg segmental angular momentum cancellation. Instead, the paired PC_BoS_ only revealed how the segments within the swing leg covaried during the perturbation response. Overall, coordination patterns extracted from angular momentum relative to different reference points provide distinctive yet complementary information about how people coordinate their body segments in response to unexpected perturbations.

One advantage of referencing angular momentum to the CoM is that one can easily identify the degree of segmental angular momentum cancellation during walking and perfect segmental cancellation would result in zero angular momentum. This analysis could be beneficial for identifying gait asymmetries as people whose contralateral limb segments do not move in complete anti-phase usually have greater L_CoM_ than controls, presumably due to a reduction in momentum cancellation between limbs (Honda et al., 2019; Nott, Neptune & Kautz, 2014; Park et al., 2021; Vistamehr et al., 2016). Whereas, if angular momentum is referenced to the edge of BoS and one wants to estimate the degree of segmental cancellation, additional analysis is necessary. Specifically, one would need to compute the difference between L_BoS_ and the angular momentum of the body CoM about the edge of the BoS to infer the degree of segmental cancellation. A larger computed difference would correspond to less segmental cancellation. Therefore, compared to angular momentum referenced to the edge of BoS, using angular momentum referenced to the CoM could provide a more direct intuition about the degree of segmental cancellation without any extra computations.

## Conclusion

We demonstrated that computing angular momentum relative to different reference points provides complementary insights into how people reactively control balance during trip-like and slip-like treadmill induced perturbations. In the future, this analysis could be extended to people with balance deficits, such as older adults and people post-stroke. In doing so, we may be able to identify differences in reactive control coordination patterns following perturbations between healthy populations and people with a higher fall risk, and this may provide more insights into how intersegmental dynamics contribute to impaired balance control during walking.

## Additional Information and Declarations

### Data Availability

The following information was supplied regarding data availability: The raw data are available at OSF: Liu, Chang, James M Finley, and Sungwoo Park. 2022. “Data.” OSF. April 15. osf.io/d7p4g.
